# Identification of Liver Immune Microenvironment-Related Hub Genes in Liver of Biliary Atresia

**DOI:** 10.3389/fped.2021.786422

**Published:** 2022-01-17

**Authors:** Jiaxu Zhang, Yi Luo, Mingxuan Feng, Qiang Xia

**Affiliations:** ^1^Department of Liver Surgery, Ren Ji Hospital, Shanghai Jiao Tong University School of Medicine, Shanghai, China; ^2^Shanghai Institute of Transplantation, Shanghai, China

**Keywords:** biliary atresia, liver autoimmune disease, immune microenvironment, prognosis, gene expression profile

## Abstract

**Background:** Biliary atresia (BA) is one of the most common and fatal abnormalities of newborns. Increasing evidences indicated that immunology was the critical part of the etiology. This research used a public gene expression database to explore the immune microenvironment of BA liver.

**Methods:** The gene expression profiles GSE46960, GSE159720, and GSE15235, containing BA and normal liver gene expression data, were obtained from the Expression Omnibus Gene. We applied CIBERSORTx to quantify 22 subsets of immune cells in BA liver. The differentially expressed genes (DEGs) and immune cells were used to further explore their relationship with liver fibrosis and the inflammation status of BA.

**Results:** The expression of immune-related genes *CXCL6, CXCL8, CXCL10, CCL20, IL32, TGFB2, SPP1*, and *SLIT2* was significantly different between BA and normal liver, among which *CXCL8* was the hub gene. Six of 22 immune cell proportions were significantly different between BA and normal liver. Specifically, M0 macrophages and resting memory CD4+ T cells were upregulated in BA liver compared with normal liver. Meanwhile, monocytes, resting natural killer (NK) cells, plasma cells, and regulatory T (Treg) cells were downregulated. A further correlation analysis revealed that *SLIT2* and *CXCL6* owned high positive correlation coefficients with fibrosis grade, while the proportion of resting NK cells was negatively correlated. Proportions of resting CD4+ memory T cells were strongly related to the inflammation grade of BA liver.

**Conclusion:** Biliary atresia is a disease strongly correlated with immune response. Our results might provide a clue for further exploration of BA etiology, which may promote a potential prediction model based on immune infiltration features.

## Introduction

Biliary atresia (BA) is one of the most common and fatal abnormalities of newborns. Most BA patients cannot survive with their native liver because of severe cholestatic cirrhosis, unless they receive Kasai portoenterostomy to reconstruct the bile drainage (1). However, more than half of the patients cannot survive with their native liver for a long term after receiving Kasai portoenterostomy, which contributes to 75% of pediatric liver transplantation under 2 years old in the world (2).

Although the etiology of BA is still not clear, some theories were related to genetics (3), immunology (4), and toxicology (5). Increasing evidence indicated that immunologic derangement was the core issue that caused BA. The animal model of BA was based on the immune reaction of rhesus rotavirus (RRV) infection in newborn Balb/c mice (6). The abnormality of B cells, T cells, and macrophages in BA patients was also revealed by single-cell RNA-seq in a small population recently, which provided strong evidence that the immune system took part in the occurrence and development of BA to a great extent (7). Therefore, research with a larger sample size were necessary to further prove these findings.

In the past, microarrays and RNA-seq data could only reflect the mRNA expressions of bulk liver tissues composed of hepatocytes, cholangiocytes, endothelial cells, and immune cells, making it impossible to demonstrate the potential functions of each kind of cell. With the development of bioinformatics, powerful deconvolution algorithms now enable us to calculate the immune microenvironment precisely by using the bulk mRNA expression data. Here, we used CIBERSORTx, a tool verified by fluorescence-activated cell sorting, to reveal the relationship between the immune microenvironment of BA liver and the progression of the disease by utilizing the present mRNA expressions data sets of BA (8). Since Rohr-Udilova et al. (9) first used it in exploring the immune cell landscape between healthy liver and hepatocellular carcinoma, CIBERSORT was widely applied in various liver diseases (10–12).

## Methods

### Data Source

Gene expression data GSE46960 (13), GSE159720 (3), and GSE15235 (14) were obtained from the “Gene Expression Omnibus” (GEO) database. The former two data series contained genetic expression profiles of BA liver and normal liver. GSE46960 was constructed by Affymetrix Human Gene 1.0 ST Array. It received liver biopsy samples from 64 infants with BA at the time of intraoperative cholangiogram and seven deceased donor children. GSE159720 was established by Illumina NextSeq 500. Only four BA samples together with three normal samples were found in this data series. GSE15235 was a data series without genetic information of normal liver. However, it had a specific grade of liver inflammation and fibrosis corresponding to the genetic expression, making it possible to analyze the correlation between liver inflammation and fibrosis with the hub genes and liver immune microenvironment.

### Data Processing and Analysis of Differentially Expressed Genes

After the original data were obtained, the raw data sets were processed according to the platforms of the chip. GSE46960 was processed by the “affy” package first, and the “Limma” package was applied in R to screen the differentially expressed genes (DEGs) of BA liver and normal liver with *P*-value < 0.05 and |log2 (fold change)| ≥ 1.2. GSE159720 was processed by the “oligo” package first, followed by the “DESeq” package in R, and the DEGs were identified under the same criteria as GSE46960. A Venn diagram of DEGs from the former data sets and ImmPort, a data set of genes related to the immune system (15), was made to explore the overlapping genes associated with the immune system.

### Functional Enrichment Analyses of DEGs in BA

The DEGs of each dataset were processed by g:profiler (https://biit.cs.ut.ee/gprofiler/gost), an online tool for functional enrichment analyses. Kyoto Encyclopedia of Genes and Genomes (KEGG) pathway and Gene Ontology (GO) pathway were selected for enrichment (16). Metascape (https://metascape.org) was an online tool to analyze the DEGs from multiple data sets (17). The enrichment analysis of DEGs from GSE46960 and GSE159720 was processed by Metascape, which exported the most significant GO and KEGG pathways in both data sets.

### Generating the Protein–Protein Interaction Network and Identification of Hub Genes

The Search Tool for the Retrieval of Interacting Genes (STRING, https://string-db.org) is a biological resource that provides systematic screens of human–protein interactions (18). The overlapping DEGs of GSE46960 and GSE159720 were processed by STRING to get a protein–protein interaction (PPI) network, after which Cytoscape was used to generate a visualized PPI network. Hub genes were identified by CytoHuba, a tool of Cytoscape, with default parameters, through which it turned out that immune-related genes were critical during the disease process.

### Liver Immune Microenvironment Analysis

CIBERSORTx (https://cibersortx.stanford.edu/) is an analytical tool to provide an estimation of the abundances of member cell types in bulk tissue expression profiles, which is widely used in immune microenvironment analysis (8). CIBERSORTx algorithm under batch mode was applied to compute the immune cell fraction in liver biopsy samples, utilizing the LM22, a validated leukocyte gene signature matrix, as the reference. The differences of immune cell fraction between BA liver and normal liver were analyzed by *t*-test. A further correlation analysis was evaluated by Pearson correlation coefficient, and *P*-value under 0.05 was considered significant.

### Exploring the Correlation of Liver Status and Immune Microenvironment

The matched liver inflammation and fibrosis grades in GSE15235 were obtained from a previous study (14). In brief, inflammation grades were assessed by hematoxylin/eosin stain. According to the hematoxylin/eosin stain, no inflammation was considered grade 0, while portal expansion together with brisk inflammation in >50% portal tracts was considered grade 3. Gomori trichrome stain was used to assess the fibrosis grade, and no fibrosis was rated as stage 0, while portal fibrosis with expansion and bridging in >50% portal tracts or regenerative nodule was rated as stage 3. All the grades can be accessed in the previous study (14). After utilizing CIBERSORTx to decode the immune cell fractions in GSE15235, the expression of overlapping genes generated by the Venn diagram and the immune cell fractions were included in the correlation analysis. The Spearman correlation coefficient evaluated the correlations, and *P*-value under 0.05 was considered significant.

## Results

### Identification of DEGs in BA

**Figure 1:**
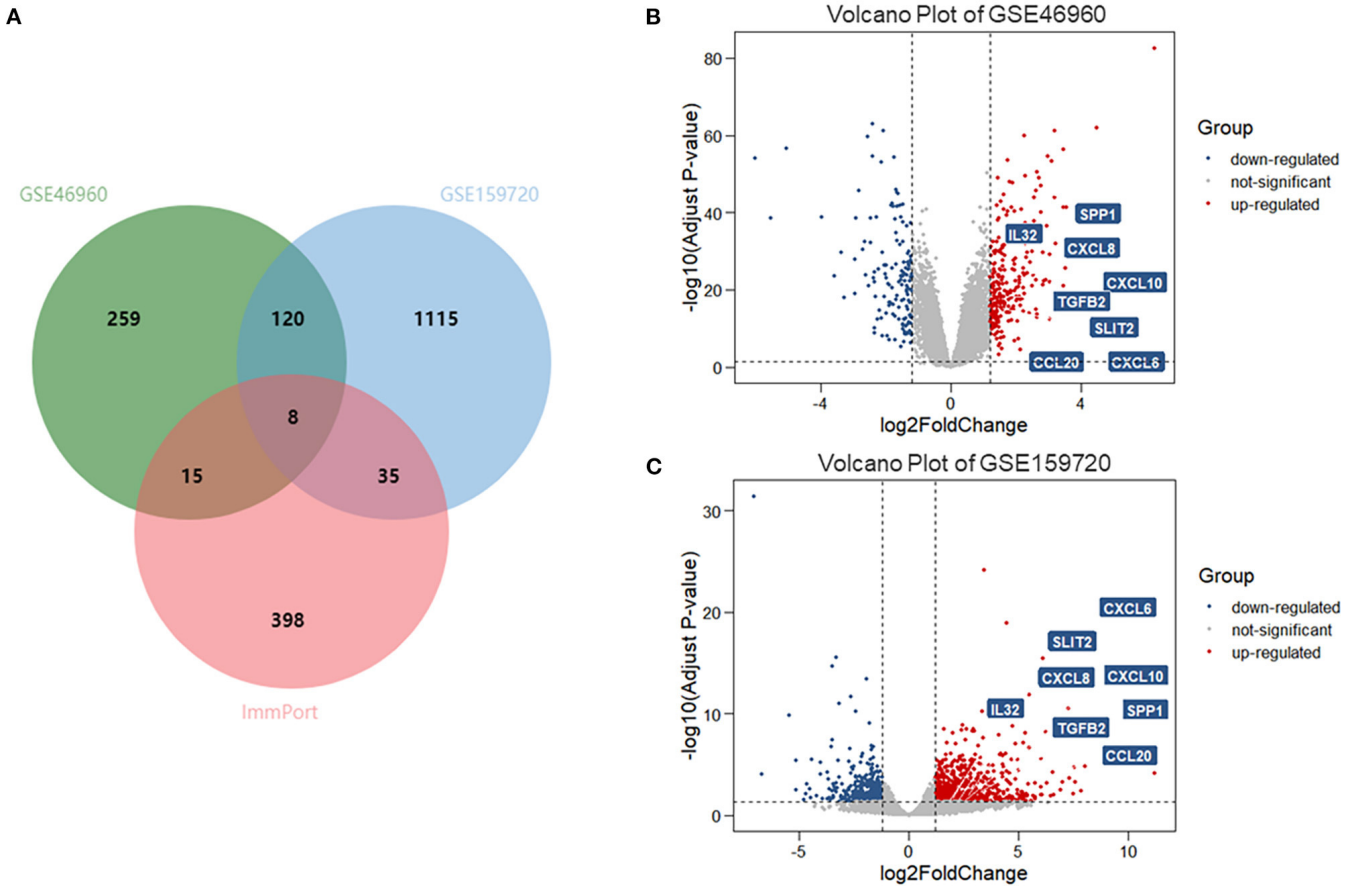
DEGs and immune-related genes in GSE46960 and GSE159720. **(A)** One hundred twenty-eight DEGs were duplicated in GSE46960 and GSE159720, among which eight DEGs were included in ImmPort. **(B,C)** Volcano plot of GSE46960 and GSE159720. The eight immune-related genes are highlighted. DEGs: differentially expressed genes.

In data set GSE46960, 402 DEGs were identified by limma test, satisfying the criterion of |log2FC| ≥ 1.2 and *P*-value < 0.05. Under the same criterion, 1,278 DEGs were discovered by DESeq test in GSE159720. By comparing the DEGs in the two data sets, the expression of 128 genes was significantly changed in both data sets. Eight of the 128 duplicated genes were found in ImmPort, suggesting that these DEGs were related to the immune process of BA (Figure 1A). The volcano plots showed the DEGs in each data set (Figures 1B,C), and eight immune-related genes are introduced in Table 1.

**Table 1:** List of eight immune-related differentially expressed genes in biliary atresia (BA) and their functions in the progress of BA.

**Gene symbol**	**Gene name**	**Origin**	**Function in BA**
CXCL8 (19)	C-X-C motif chemokine ligand 8	BECs	Promoting ductular reaction and associated liver fibrogenesis
CXCL6 (20)	C-X-C motif chemokine ligand 6	Unknown	Stimulating Kupffer cells releasing TGF-β and activating stellate cells
CXCL10 (21)	C-X-C motif chemokine ligand 10	BECs	Stimulation of monocytes, natural killer, and T-cell migration
CCL20 (22)	C-C motif chemokine ligand 20	BECs	Th17 cell recruitment
IL32 (23)	Interleukin 32	BECs	Amplification and continuance of periductal inflammatory reactions
SLIT2 (24, 25)	Slit guidance ligand 2	Unknown	Activating hepatic stellate cells
SPP1 (26)	Secreted phosphoprotein 1	BECs	Stimulating T-cell proliferation and inducing T-cells and macrophages to express other Th1 cytokines
TGFB2 (27, 28)	Transforming growth factor beta 2	BECs, hepatocytes, and mesenchymal cells	Unknown

*BECs, biliary epithelial cells*.

### Functional Enrichment for BA

**Figure 2:**
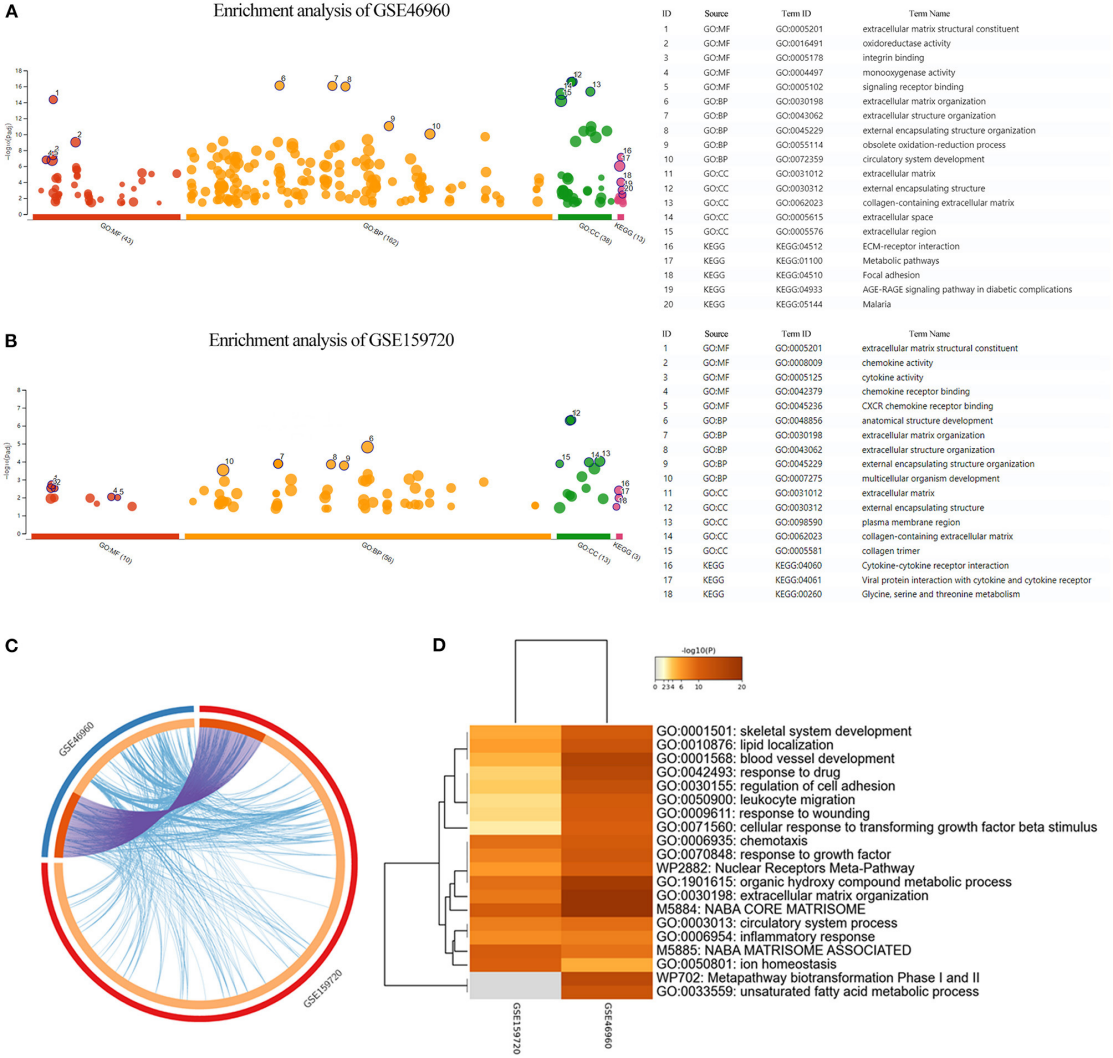
Enrichment analysis of GEO data sets. **(A,B)** GO enrichment analysis and KEGG enrichment analysis of GSE46960 and GSE159720 demonstrated that the immune process plays an essential role in biliary atresia liver according to gene expression. **(C)** Overview of duplicate DEGs and shared ontology terms of GSE46960 and GSE159720. Purple lines linked the duplicated genes, and blue lines linked the different genes where they fall into the same ontology term. **(D)** Heat map of enriched terms across GSE46960 and GSE159720. Here, we visualized the top 20 clusters. GO, Gene Ontology; MF, molecular function; BP, biological process; CC, cellular component; KEGG, Kyoto Encyclopedia of Genes and Genomes.

Differentially expressed genes in the datasets were selected to perform KEGG and GO functional enrichment analyses, utilizing the g:profiler tool to explore the biological effects, as shown in Figures 2A,B. In GSE46960, the pathways were mainly focused on extracellular matrix in both GO and KEGG enrichment. In GSE159720, besides the pathways related to the extracellular matrix, more immune-related functions were enriched, such as chemokine activity, cytokine activity, and chemokine receptor binding. Figure 2C demonstrates the same genes that are shared by both GSE46960 and GSE159720 with purple arches, and the blue lines link the different genes, where they fall into the same ontology term with *p* < 0.05. To further explore the pathways that were critical in both gene sets, a functional enrichment analysis, which took consideration of GSE46960 and GSE159720 at the same time, was performed by Metascape (Figure 2D). Pathways of leukocyte migration, chemotaxis, and inflammatory response were in the top 20 clusters of the enriched pathways.

### Hub Genes of BA

**Figure 3:**
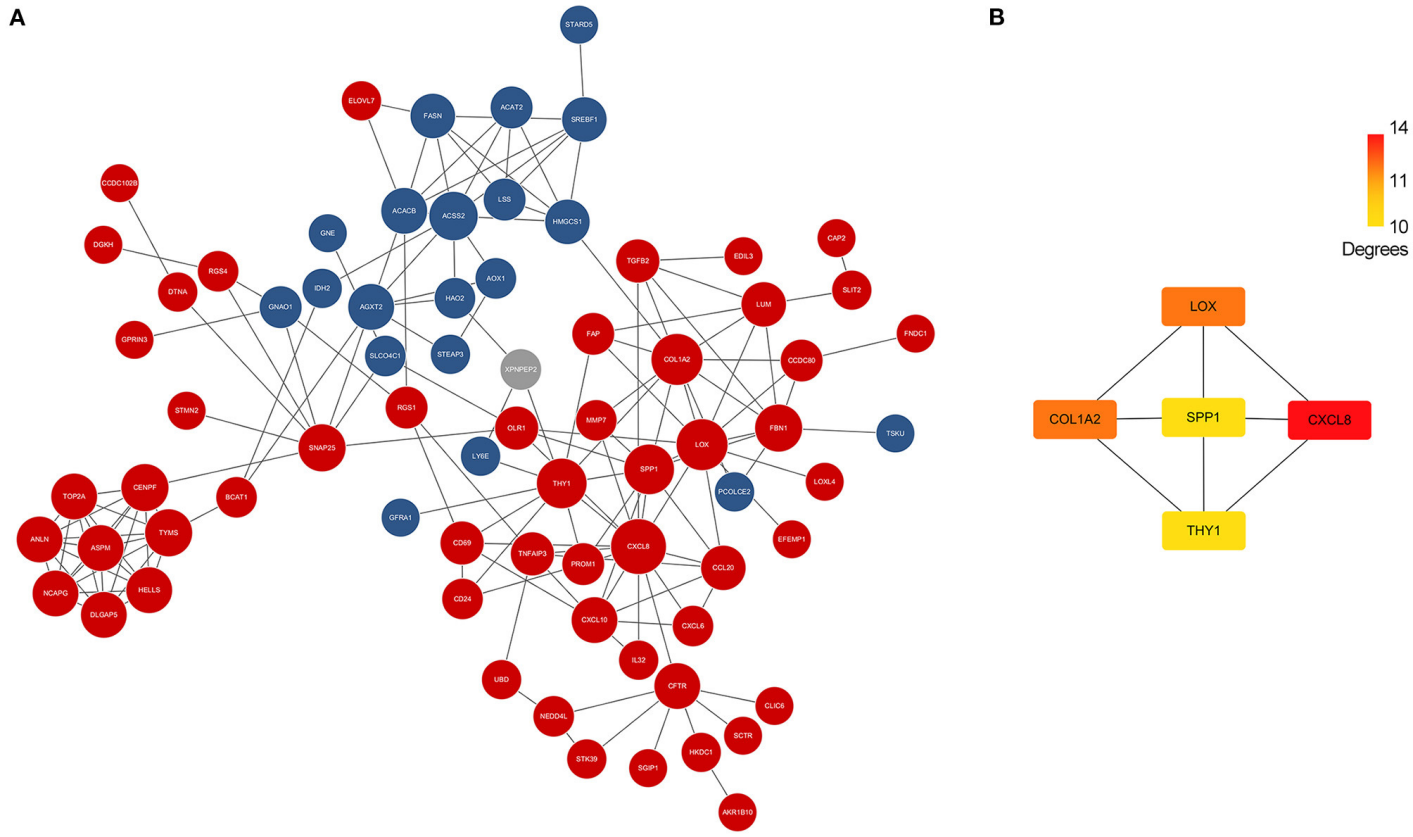
PPI network of DEGs and hub genes. **(A)** PPI network of DEGs in GSE46960 and GSE159720. Red spots and blue spots represented upregulated genes and downregulated genes in both data sets, respectively, while gray spots represented an inconsistency of expression in each data set. **(B)** Hub genes identified by Cytohubba. *CXCL8* was the top hub gene with 14 degrees, followed by *COL1A2, LOX, SPP1*, and *THY1*. PPI, protein–protein interaction; DEGs, differentially expressed genes.

Differentially expressed genes which overlapped in GSE46960 and GSE159720 were analyzed in STRING to evaluate the interaction between these genes further, and a total of 85 nodes and 163 edges were identified from the PPI network; 11 disconnected nodes in the network were hidden, as shown in Figure 3A. CytoHubba identified the top five hub genes according to the degree algorithms (Figure 3B), and *CXCL8* was ranked as number one with 14 degrees, proving that the immune system may play a considerable role in the pathophysiology of BA.

### Immune Microenvironment of BA Liver

**Figure 4:**
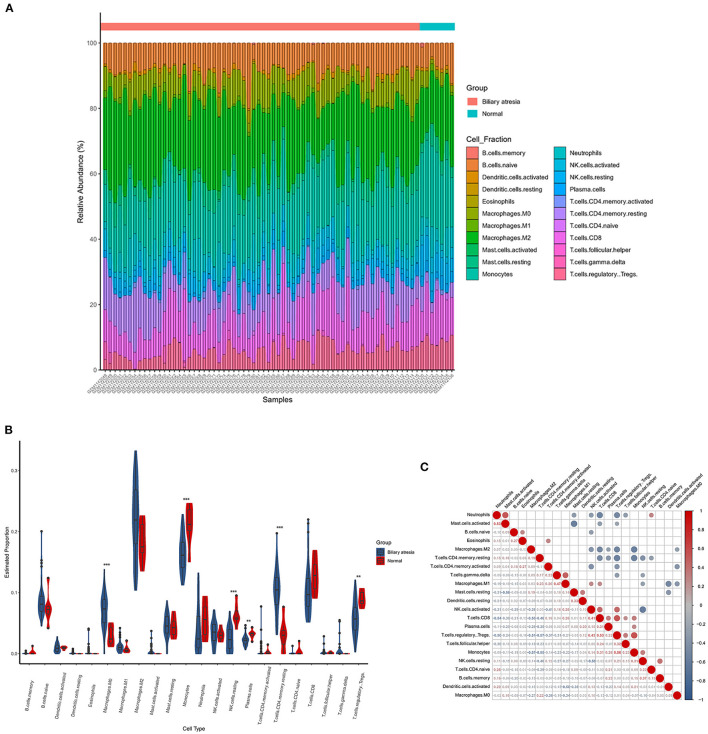
The landscape of immune infiltration between biliary atresia (BA) and normal controls. **(A)** The box plot diagram indicated the relative percentage of different types of immune cells. **(B)** The violin plot demonstrated the difference of immune infiltration between BA (blue) and normal (red) controls. **(C)** The correlation matrix of immune cell proportion in GSE46960.

The CIBERSORTx deconvolution algorithm was applied to access the immune cell compositions of BA liver according to the GSE46960 dataset. Figure 4A summarized the 22 kinds of immune cell compositions from 64 BA liver and seven normal livers. Six of the 22 immune cell proportions were significantly different between BA and normal liver (Figure 4B). As shown in Figure 4B, M0 macrophages and resting memory CD4+ T cells were upregulated in BA liver compared with normal liver. Monocytes, resting natural killer (NK) cells, plasma cells, and regulatory T (Treg) cells were downregulated. The results of the correlation analysis between LM22 (Figure 4C) found that monocytes and Treg cells have the most intense positive relationship with *r* = 0.56 (*p* < 0.05). Except for the strong negative relationship between all kinds of resting cells and activated cells, Treg cells, and resting memory CD4+ T cells had the highest negative correlation coefficients with *r* = −0.57 (*p* < 0.05).

### Correlation Between Liver Immune Microenvironment and Disease Status

**Figure 5:**
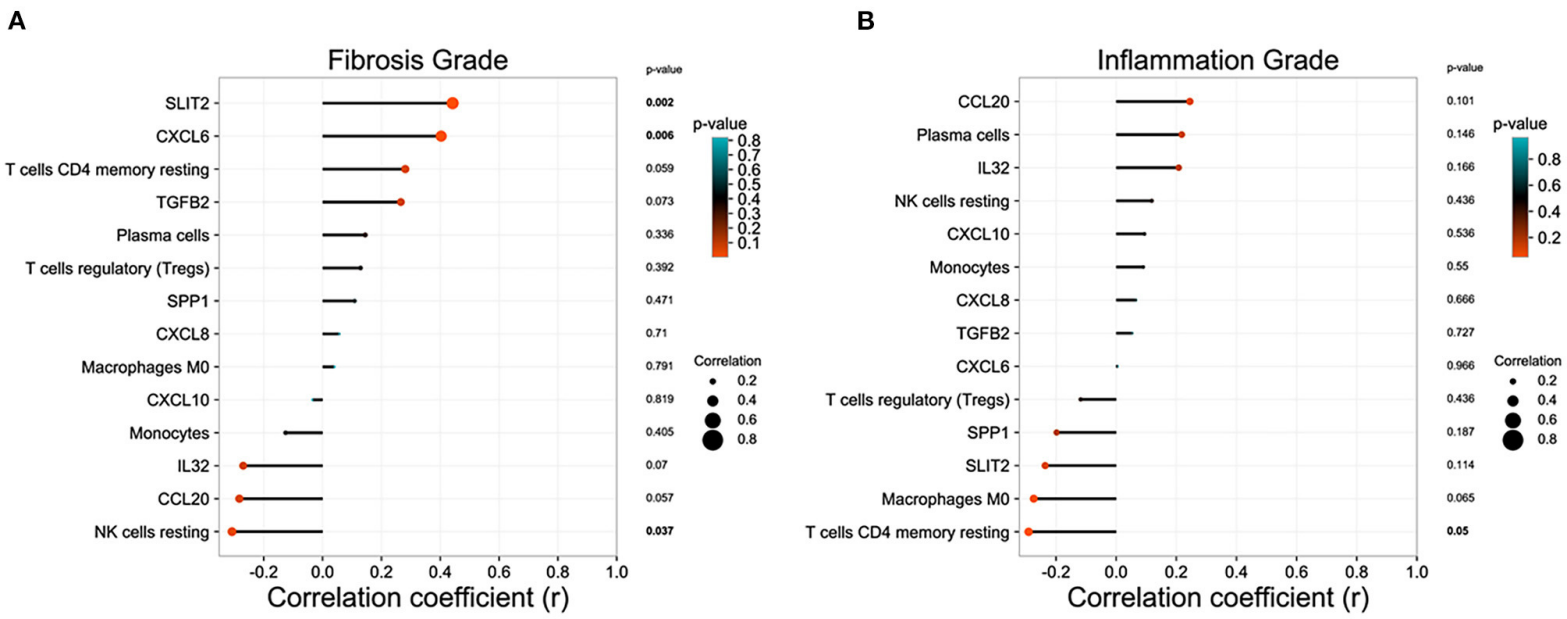
Correlation analysis between the immune microenvironment and liver status in biliary atresia (BA). **(A)** Relationship between immune microenvironment and liver fibrosis grade in BA liver. **(B)** Relationship between immune microenvironment and liver inflammation grade in BA liver.

In GSE15235, we found 47 BA patients with clinical information, such as survival time of native liver, liver function, and the pathology grade of liver inflammation and fibrosis. After excluding patients without pathology grades of liver inflammation and fibrosis, 46 BA patients were included in the correlation analysis. Eight DEGs relating to immune system and immune cell proportions that were significantly regulated in BA liver were incorporated into the analysis to figure out the relationship between these parameters and the pathology grade of liver inflammation and fibrosis. *SLIT2* and *CXCL6* own high positive correlation coefficients with fibrosis grade, while the proportion of resting NK cells was negatively correlated (Figure 5A). The proportions of resting CD4+ memory T cells are strongly related to the inflammation grade of BA liver (Figure 5B).

## Discussion

Biliary atresia is a fatal neonatal cholestatic liver disease with unknown etiology. However, sights were increasingly focused on the immune response to figure this puzzle out. Recently, Wang et al. (7) and Taylor et al. (29) used single-cell RNA sequencing to demonstrate the abnormality of immune cells in BA patients, which further proved the role of immune response in BA pathogenesis. Here, we utilized the public gene expression data set and deconvolution algorithm of BA liver to explore its immune-related genes and immune microenvironment. In this study, eight immune-related DEGs and six subtypes of immune cells were identified as critical factors in the progression of the disease.

Most of the immune-related DEGs that we found in this study were chemokines, which may greatly contribute to the pathogenesis of the BA. *CXCL8*, a chemotactic factor that guides neutrophils to the site of infection, was identified as the hub gene of BA in this study. The expression of *CXCL8* was high in liver and peripheral blood in BA patients. In peripheral blood, it mainly originated from monocytes, neutrophils, T cells, and B cells (30). Meanwhile, it was primarily expressed in the hepatic parenchyma in liver (31). *CXCL8* in BA liver was predominantly in cholangiocytes within areas of ductular reaction, which may play an essential role in BA progress by mediating the ductular reaction and liver fibrogenesis (19). It was believed that both expressions of *CXCL8* in the liver and peripheral blood were a predominant part in BA progress and can be used as prognosis predictors of BA patients (32).

*SLIT2* is an immune-related gene that regulates cell growth and migration (24). It can recruit immune cells, such as macrophages (33, 34). However, the mechanism may be different in the liver. Former studies found that liver fibrosis was mediated by activating hepatic stellate cells through the Slit2/Robo1 and Slit2/Robo2 signal pathway (24, 25). It has been proved that the hepatic expression of *SLIT2* was significantly increased in patients with primary biliary cirrhosis and in bile duct ligation mouse model (35). CXCL6 is a chemokine which can recruit neutrophil, leading to tissue damage and prolonged inflammatory responses (36). Recently, CXCL6 was found to be upregulated in the serum and liver tissue of high-stage liver fibrosis patients (20) and was supposed to participate in fibrogenesis by stimulating Kupffer cells releasing TGF-β and thereby activating stellate cells. This study finds that the expression of *SLIT2* and *CXCL6* in BA liver is exceptionally high, and it is strongly related to liver fibrosis grade.

The microenvironment of BA was identified by CIBERSORTx. Natural killer cells, monocytes, macrophages, Treg cells, and plasma cells were significantly different in BA and normal livers, as reported in previous research. Moreover, a more specific fraction of immune cells was computed, and correlation analysis was applied to figure out the relationship between microenvironment and liver status of BA patients.

Natural killer cells were found to have adverse effects in BA livers, especially in experimental BA (37–39). Natural killer cells can be activated by dendritic cells in the RRV BA model, thus injuring the biliary trees (40). The deletion of NK cells further proved that the activation of NK cells plays an essential role in bile duct injury (38). Alexandra et al. (41) supposed that *CXCL9* and *CXCL10* were secreted by cholangiocytes after a virus infection. Natural killer cells were activated *via* NKG2D ligands expressed by infected cholangiocytes and migrated to the liver and biliary tree along the chemokine gradient of CXCL9 and CXCL10, causing an autoimmune response against biliary epithelium (41). In our research, although the proportion of NK cells activated is similar between normal liver and BA liver, the proportion of NK cells resting is significantly decreased in BA liver. This phenomenon may be caused by NK cells exhausting after immune response in BA liver. The correlation analysis of this research also revealed that NK cells resting was negatively correlated with chronic fibrosis of BA liver in humans, as reported formerly (42), which may give a clue in retarding the fibrosis procedure in BA patients. The inflammation grade of BA liver in our study reflects an acute inflammation status of BA liver. The positive correlation between inflammation grade and NK cell resting may reflect the dysfunction of NK cells in killing activated T cells and other liver-resident cells and promote the inflammation response (43).

The proportion of Treg cells was severely reduced according to our research. Treg cells play an essential role in regulating the immune microenvironment and maintaining immune homeostasis (44). In the murine BA model established by RRV, liver Treg cells decreased in both number and function (45), contributing to the progress of BA. It was proved that the proportion of Treg cells in peripheral blood was reduced in BA patients who were positive for cytomegalovirus (46). Although we found that the proportion of Treg cells was significantly reduced in BA liver, the correlation analysis did not detect the relationship between Treg cells and liver fibrosis grade or liver inflammation grade. On the other hand, the correlation analysis revealed that Treg cells had the widest connection with other immune cells. Both the strongest positive and negative correlation coefficients were related to the Treg cells, reflecting its powerful regulation role in BA liver.

The mononuclear phagocyte system, including macrophages and monocytes, was suppressed in BA liver. Although the total amount of macrophage was significantly high in BA liver, the subtypes of functional macrophages, such as M1 and M2 macrophage, did not show any statistical difference. Previous studies did not have a consensus on the effect of macrophage in BA progression (47–49). The proportion of M0 macrophage in BA liver was remarkably higher than the one in normal liver, indicating that macrophages were inactivated. Besides this, the proportion of monocytes was significantly decreased in BA liver, further demonstrating that the mononuclear phagocyte system was dysfunctional. As Wang et al. (7) have reported, the macrophages in BA liver were under a hypo-inflammation situation. Meanwhile, the Kupffer cell scavenger function was defective, which may explain why the proportion of M0 macrophages was extremely increased in BA liver.

In this research, we applied CIBERSORTx in the exploration of the microenvironment and correlation between immune cell infiltration and BA status. Meanwhile, several limitations inevitably existed in our exploration. Firstly, this result was calculated by the algorithm, which was an estimation of the actual situation. Despite the fact that LM22, the reference signature of immune cells, was widely used in CIBERSORT-related analysis, including liver diseases, no study has validated the reliability of LM22 in liver research, which may misrepresent the immune cell types present and active in BA or normal livers. Besides this, the present research cohort is limited; a prospective was required to validate the results. In any case, the correlation between immune cell infiltration and BA status not only deserves further research but also provides a potential prediction tool.

## Conclusion

In conclusion, we depicted the microenvironment of BA liver and the correlation between the microenvironment and patient status. The expression of *CXCL6, CXCL8, CXCL10, CCL20, IL32, TGFB2, SPP1*, and *SLIT2* was significantly different between BA and normal liver. The abnormal accumulation of six types of immune cells played an essential role in BA, such as the high proportions of M0 macrophages and resting memory CD4+ T cells and the low proportions of monocytes, resting NK cells, plasma cells, and Treg cells. Our results might provide a clue for further exploration of BA etiology and promote a potential prediction model based on immune infiltration features.

## Data Availability Statement

The original contributions presented in the study are included in the article/supplementary material, further inquiries can be directed to the corresponding author/s.

## Author Contributions

QX, YL, and JZ designed this research. JZ and MF collected data and wrote the manuscript. JZ and YL analyzed data. All authors read and approved the final manuscript.

## Funding

This work was supported by grants from the National Natural Science Foundation of China (grant nos.: 81972205 and 92059205) and Shenkang three-year program (grant nos.: SHDC2020CR5012 and SHDC2020CR2003A).

## Conflict of Interest

The authors declare that the research was conducted in the absence of any commercial or financial relationships that could be construed as a potential conflict of interest.

## Publisher's Note

All claims expressed in this article are solely those of the authors and do not necessarily represent those of their affiliated organizations, or those of the publisher, the editors and the reviewers. Any product that may be evaluated in this article, or claim that may be made by its manufacturer, is not guaranteed or endorsed by the publisher.

## References

[1] Harpavat S Garcia-Prats JA Anaya C Brandt ML Lupo PJ Finegold MJ. Diagnostic yield of newborn screening for biliary atresia using direct or conjugated bilirubin measurements. JAMA. (2020) 323:1141–50. 10.1001/jama.2020.0837 32207797 PMC7093763

[2] Bezerra JA Wells RG Mack CL Karpen SJ Hoofnagle JH Doo E. Biliary atresia: clinical and research challenges for the twenty-first century. Hepatology. (2018) 68:1163–73. 10.1002/hep.29905 29604222 PMC6167205

[3] So J Ningappa M Glessner J Min J Ashokkumar C Ranganathan S. Biliary-atresia-associated mannosidase-1-alpha-2 gene regulates biliary and ciliary morphogenesis and laterality. Front Physiol. (2020) 11:538701. 10.3389/fphys.2020.538701 33192543 PMC7662016

[4] Ortiz-Perez A Donnelly B Temple H Tiao G Bansal R Mohanty SK. Innate immunity and pathogenesis of biliary atresia. Front Immunol. (2020) 11:329. 10.3389/fimmu.2020.00329 32161597 PMC7052372

[5] Waisbourd-Zinman O Koh H Tsai S Lavrut PM Dang C Zhao X. The toxin biliatresone causes mouse extrahepatic cholangiocyte damage and fibrosis through decreased glutathione and SOX17. Hepatology. (2016) 64:880–93. 10.1002/hep.28599 27081925 PMC4992464

[6] Mohanty SK Lobeck I Donnelly B Dupree P Walther A Mowery S. Rotavirus reassortant-induced murine model of liver fibrosis parallels human biliary atresia. Hepatology. (2020) 71:1316–30. 10.1002/hep.30907 31442322 PMC7384231

[7] Wang J Xu Y Chen Z Liang J Lin Z Liang H. Liver immune profiling reveals pathogenesis and therapeutics for biliary atresia. Cell. (2020) 183:1867.e26–83.e26. 10.1016/j.cell.2020.10.048 33248023

[8] Newman AM Steen CB Liu CL Gentles AJ Chaudhuri AA Scherer F. Determining cell type abundance and expression from bulk tissues with digital cytometry. Nat Biotechnol. (2019) 37:773–82. 10.1038/s41587-019-0114-2 31061481 PMC6610714

[9] Rohr-Udilova N Klinglmuller F Schulte-Hermann R Stift J Herac M Salzmann M. Deviations of the immune cell landscape between healthy liver and hepatocellular carcinoma. Sci Rep. (2018) 8:6220. 10.1038/s41598-018-24437-5 29670256 PMC5906687

[10] He Y Zhou Y Wang H Yin J Chang Y Hu P. Identifying potential biomarkers in hepatitis B virus infection and its response to the antiviral therapy by integrated bioinformatic analysis. J Cell Mol Med. (2021) 25:6558–72. 10.1111/jcmm.16655 34041839 PMC8278120

[11] Yu K Yang J Xie W Wu F Wang M Li N. Integrated bioinformatic analysis revealed biological processes and immune cells implicated in autoimmune hepatitis. J Cell Physiol. (2021) 236:5411–20. 10.1002/jcp.30246 33595095

[12] Zhang Y Chen S Li J Dai W Qian Y. Immune infiltrating cells in cholangiocarcinoma may become clinical diagnostic markers: based on bioinformatics analysis. World J Surg Oncol. (2021) 19:59. 10.1186/s12957-021-02168-8 33618734 PMC7901112

[13] Bessho K Mourya R Shivakumar P Walters S Magee JC Rao M. Gene expression signature for biliary atresia and a role for interleukin-8 in pathogenesis of experimental disease. Hepatology. (2014) 60:211–23. 10.1002/hep.27045 24493287 PMC4077977

[14] Moyer K Kaimal V Pacheco C Mourya R Xu H Shivakumar P. Staging of biliary atresia at diagnosis by molecular profiling of the liver. Genome Med. (2010) 2:33. 10.1186/gm154 20465800 PMC2887077

[15] Bhattacharya S Andorf S Gomes L Dunn P Schaefer H Pontius J. ImmPort: disseminating data to the public for the future of immunology. Immunol Res. (2014) 58:234–9. 10.1007/s12026-014-8516-1 24791905

[16] Raudvere U Kolberg L Kuzmin I Arak T Adler P Peterson H. g:Profiler: a web server for functional enrichment analysis and conversions of gene lists (2019 update). Nucleic Acids Res. (2019) 47:W191–8. 10.1093/nar/gkz369 31066453 PMC6602461

[17] Zhou Y Zhou B Pache L Chang M Khodabakhshi AH Tanaseichuk O. Metascape provides a biologist-oriented resource for the analysis of systems-level datasets. Nat Commun. (2019) 10:1523. 10.1038/s41467-019-09234-6 30944313 PMC6447622

[18] Von Mering C Huynen M Jaeggi D Schmidt S Bork P Snel B. STRING: a database of predicted functional associations between proteins. Nucleic Acids Res. (2003) 31:258–61. 10.1093/nar/gkg034 12519996 PMC165481

[19] Godbole N Nyholm I Hukkinen M Davidson JR Tyraskis A Eloranta K. Prognostic and pathophysiologic significance of IL-8 (CXCL8) in biliary atresia. J Clin Med. (2021) 10:2705. 10.3390/jcm10122705 34207442 PMC8234515

[20] Cai X Li Z Zhang Q Qu Y Xu M Wan X. CXCL6-EGFR-induced Kupffer cells secrete TGF-beta1 promoting hepatic stellate cell activation via the SMAD2/BRD4/C-MYC/EZH2 pathway in liver fibrosis. J Cell Mol Med. (2018) 22:5050–61. 10.1111/jcmm.13787 30106235 PMC6156397

[21] Koniaris LG Zimmers-Koniaris T Hsiao EC Chavin K Sitzmann JV Farber JM. Cytokine-responsive gene-2/IFN-inducible protein-10 expression in multiple models of liver and bile duct injury suggests a role in tissue regeneration. J Immunol. (2001) 167:399–406. 10.4049/jimmunol.167.1.399 11418676

[22] Chen P Zhong Z Jiang H Chen H Lyu J Zhou L. Th17-associated cytokines multiplex testing indicates the potential of macrophage inflammatory protein-3 alpha in the diagnosis of biliary atresia. Cytokine. (2019) 116:21–6. 10.1016/j.cyto.2019.01.002 30684914

[23] Okamura A Harada K Nio M Nakanuma Y. Interleukin-32 production associated with biliary innate immunity and proinflammatory cytokines contributes to the pathogenesis of cholangitis in biliary atresia. Clin Exp Immunol. (2013) 173:268–75. 10.1111/cei.12103 23607494 PMC3722927

[24] Chang J Lan T Li C Ji X Zheng L Gou H. Activation of Slit2-Robo1 signaling promotes liver fibrosis. J Hepatol. (2015) 63:1413–20. 10.1016/j.jhep.2015.07.033 26264936

[25] Zeng Z Wu Y Cao Y Yuan Z Zhang Y Zhang DY. Slit2-Robo2 signaling modulates the fibrogenic activity and migration of hepatic stellate cells. Life Sci. (2018) 203:39–47. 10.1016/j.lfs.2018.04.017 29660433 PMC6322547

[26] Whitington PF Malladi P Melin-Aldana H Azzam R Mack CL Sahai A. Expression of osteopontin correlates with portal biliary proliferation and fibrosis in biliary atresia. Pediatr Res. (2005) 57:837–44. 10.1203/01.PDR.0000161414.99181.61 15845635

[27] Lee SY Chuang JH Huang CC Chou MH Wu CL Chen CM. Identification of transforming growth factors actively transcribed during the progress of liver fibrosis in biliary atresia. J Pediatr Surg. (2004) 39:702–8. 10.1016/j.jpedsurg.2004.01.030 15137003

[28] Kerola A Lohi J Heikkila P Mutanen A Jalanko H Pakarinen MP. Divergent expression of liver transforming growth factor superfamily cytokines after successful portoenterostomy in biliary atresia. Surgery. (2019) 165:905–11. 10.1016/j.surg.2018.12.003 30686515

[29] Taylor SA Chen SY Gadhvi G Feng L Gromer KD Abdala-Valencia H. Transcriptional profiling of pediatric cholestatic livers identifies three distinct macrophage populations. PLoS ONE. (2021) 16:e0244743. 10.1371/journal.pone.0244743 33411796 PMC7790256

[30] Zhang Y Zhou L Gu G Feng M Ding X Xia Q. CXCL8(high) inflammatory B cells in the peripheral blood of patients with biliary atresia are involved in disease progression. Immunol Cell Biol. (2020) 98:682–92. 10.1111/imcb.12366 32506479

[31] Arafa RS Abdel Haie OM El-Azab DS Abdel-Rahman AM Sira MM. Significant hepatic expression of IL-2 and IL-8 in biliary atresia compared with other neonatal cholestatic disorders. Cytokine. (2016) 79:59–65. 10.1016/j.cyto.2015.12.023 26765485

[32] Dong R Zheng S. Interleukin-8: a critical chemokine in biliary atresia. J Gastroenterol Hepatol. (2015) 30:970–6. 10.1111/jgh.12900 25611432

[33] Wang L Zheng J Pathak JL Chen Y Liang D Yang L. SLIT2 overexpression in periodontitis intensifies inflammation and alveolar bone loss, possibly via the activation of MAPK pathway. Front Cell Dev Biol. (2020) 8:593. 10.3389/fcell.2020.00593 32760720 PMC7371784

[34] Geraldo LH Xu Y Jacob L Pibouin-Fragner L Rao R Maissa N. SLIT2/ROBO signaling in tumor-associated microglia/macrophages drives glioblastoma immunosuppression and vascular dysmorphia. J Clin Invest. (2021). 131:e141083. 10.1172/JCI141083 34181595 PMC8363292

[35] Li C Yang G Lin L Xuan Y Yan S Ji X. Slit2 signaling contributes to cholestatic fibrosis in mice by activation of hepatic stellate cells. Exp Cell Res. (2019) 385:111626. 10.1016/j.yexcr.2019.111626 31545977

[36] Besnard AG Struyf S Guabiraba R Fauconnier L Rouxel N Proost P. CXCL6 antibody neutralization prevents lung inflammation and fibrosis in mice in the bleomycin model. J Leukoc Biol. (2013) 94:1317–23. 10.1189/jlb.0313140 23975892

[37] Shivakumar P Sabla GE Whitington P Chougnet CA Bezerra JA. Neonatal NK cells target the mouse duct epithelium via Nkg2d and drive tissue-specific injury in experimental biliary atresia. J Clin Invest. (2009) 119:2281–90. 10.1172/JCI38879 19662681 PMC2719928

[38] Saxena V Shivakumar P Sabla G Mourya R Chougnet C Bezerra JA. Dendritic cells regulate natural killer cell activation and epithelial injury in experimental biliary atresia. Sci Transl Med. (2011) 3:102ra194. 10.1126/scitranslmed.3002069 21957172 PMC4006997

[39] Yang L Mizuochi T Shivakumar P Mourya R Luo Z Gutta S. Regulation of epithelial injury and bile duct obstruction by NLRP3, IL-1R1 in experimental biliary atresia. J Hepatol. (2018) 69:1136–44. 10.1016/j.jhep.2018.05.038 29886157 PMC6314850

[40] Miethke AG Saxena V Shivakumar P Sabla GE Simmons J Chougnet CA. Post-natal paucity of regulatory T cells and control of NK cell activation in experimental biliary atresia. J Hepatol. (2010) 52:718–26. 10.1016/j.jhep.2009.12.027 20347178 PMC2864543

[41] Sharland A Gorrell MD. Cooperation of innate and adaptive immunity in the pathogenesis of biliary atresia: there's a killer on the run. Hepatology. (2009) 50:2037–40. 10.1002/hep.23399 19937699

[42] Highton AJ Schuster IS Degli-Esposti MA Altfeld M. The role of natural killer cells in liver inflammation. Semin Immunopathol. (2021) 43:519–533. 10.1007/s00281-021-00877-6 34230995 PMC8260327

[43] Waggoner SN Cornberg M Selin LK Welsh RM. Natural killer cells act as rheostats modulating antiviral T cells. Nature. (2012) 481:394–U183. 10.1038/nature10624 22101430 PMC3539796

[44] Selck C Dominguez-Villar M. Antigen-specific regulatory T cell therapy in autoimmune diseases and transplantation. Front Immunol. (2021) 12:661875. 10.3389/fimmu.2021.661875 34054826 PMC8160309

[45] Tucker RM Feldman AG Fenner EK Mack CL. Regulatory T cells inhibit Th1 cell-mediated bile duct injury in murine biliary atresia. J Hepatol. (2013) 59:790–6. 10.1016/j.jhep.2013.05.010 23685050 PMC3855478

[46] Brindley SM Lanham AM Karrer FM Tucker RM Fontenot AP Mack CL. Cytomegalovirus-specific T-cell reactivity in biliary atresia at the time of diagnosis is associated with deficits in regulatory T cells. Hepatology. (2012) 55:1130–8. 10.1002/hep.24807 22105891 PMC3319336

[47] Davenport M Gonde C Redkar R Koukoulis G Tredger M Mieli-Vergani G. Immunohistochemistry of the liver and biliary tree in extrahepatic biliary atresia. J Pediatr Surg. (2001) 36:1017–25. 10.1053/jpsu.2001.24730 11431768

[48] Kotb MA El Henawy A Talaat S Aziz M El Tagy GH El Barbary MM. Immune-mediated liver injury: prognostic value of CD4+, CD8+, and CD68+ in infants with extrahepatic biliary atresia. J Pediatr Surg. (2005) 40:1252–7. 10.1016/j.jpedsurg.2005.05.007 16080928

[49] Yang Y Dong R Zheng C Zheng S Chen G. Infiltration of polarized macrophages associated with liver fibrosis in infants with biliary atresia. J Pediatr Surg. (2017) 52:1984–8. 10.1016/j.jpedsurg.2017.08.045 28927974

